# CORESH: a gene signature-based search engine for public gene expression datasets

**DOI:** 10.1093/nar/gkaf372

**Published:** 2025-05-05

**Authors:** Vladimir Sukhov, Aigul Nugmanova, Yury Vorontsov, Parul Mehrotra, Maksim Kleverov, Kodi Ravichandran, Maxim Artyomov, Alexey Sergushichev

**Affiliations:** Department of Pathology and Immunology, Washington University in St. Louis School of Medicine, St. Louis, MO 63110, United States; Computer Technologies Laboratory, ITMO University, Saint Petersburg 197101, Russia; Department of Pathology and Immunology, Washington University in St. Louis School of Medicine, St. Louis, MO 63110, United States; Globus Media Ltd, Moscow 115230, Russia; Kusuma School of Biological Sciences, Indian Institute of Technology Delhi, New Delhi 110016, India; Department of Pathology and Immunology, Washington University in St. Louis School of Medicine, St. Louis, MO 63110, United States; Department of Pathology and Immunology, Washington University in St. Louis School of Medicine, St. Louis, MO 63110, United States; VIB-UGent Center for Inflammation Research, Ghent, Flanders 9052, Belgium; Department of Biomedical Molecular Biology, Ghent University, Ghent, Flanders, 9052, Belgium; Department of Pathology and Immunology, Washington University in St. Louis School of Medicine, St. Louis, MO 63110, United States; Department of Pathology and Immunology, Washington University in St. Louis School of Medicine, St. Louis, MO 63110, United States; Computer Technologies Laboratory, ITMO University, Saint Petersburg 197101, Russia

## Abstract

Public data repositories like Gene Expression Omnibus (GEO) contain an extensive amount of data from hundreds of thousands of experiments, making them a valuable resource for researchers. A common scenario for utilizing this resource is to show transcriptional similarity of one’s own data to a public dataset as evidence of potentially similar biology. However, when searching for such datasets, researchers are usually limited to keyword-based search, which requires having a specific hypothesis and relies on the presence of high-quality metadata in public datasets. Here, we introduce CORESH, a web server designed to systematically find GEO datasets that match a user-provided gene signature—such as a list of top upregulated genes in response to a treatment—in a data-driven manner. CORESH operates on a compendium of >40 000 human and 40 000 mouse datasets and outputs a ranked list of datasets where the input genes exhibit similar expression patterns. The discovered datasets can then be used to identify experimental conditions associated with the activation of the query signature, offering insights into underlying biological mechanisms and guiding experimental validation. CORESH is freely accessible at https://alserglab.wustl.edu/coresh/, requires no login, and is regularly updated with the latest GEO data.

## Graphical Abstract

**Graphical Abstract:**
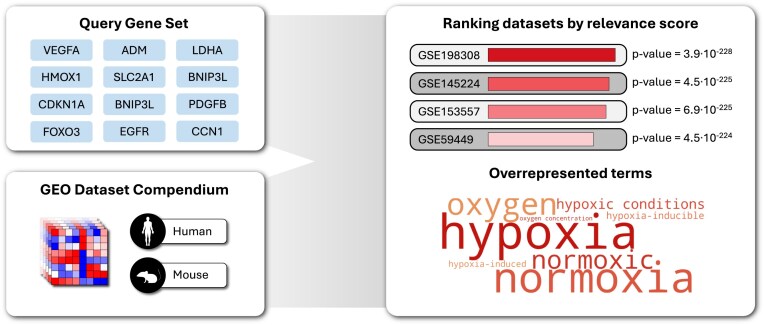

## Introduction

The Gene Expression Omnibus (GEO) is an ever-growing resource of public gene expression data, which currently contains millions of samples from hundreds of thousands of experiments [1]. This resource is highly valued by the research community for its ability to accelerate biological discovery [2], as evidenced by over 30 000 studies that have utilized previously generated GEO datasets [1]. A particularly versatile approach to using GEO involves relating transcriptional changes in a researcher’s own dataset to those in previously published experiments. These comparisons serve as a basis for generating hypotheses about the researcher’s biological system. Within this framework, GEO provides an extensive and diverse collection of experiments, such as gene perturbations, drug treatments, and profiling of tissues and cell types in both healthy and diseased conditions.

There are two general approaches for finding relevant GEO datasets: metadata-based and data-based. Metadata-based search is provided by GEO itself and is further improved by tools like ReGEO [3] or Gemma [4]. However, this approach is fundamentally limited by its hypothesis-driven nature. Conversely, data-driven search can enable users to unbiasedly find potential connections, significantly improving the comprehensiveness and usefulness of the analysis. This approach can be implemented by first creating a collection of gene signatures derived computationally from GEO datasets through differential gene expression analysis and then using this collection to find signatures that significantly overlap with a user-provided gene set. Examples include ImmuneSigDB [5] or, more recently, KnockTF [6], with both based on curated collections of GEO datasets that cover <1% of >160 000 gene expression datasets in GEO. Advances in machine learning are expanding this coverage, as seen in RummaGEO [7], which features gene signatures from around 30 000 RNA-seq datasets. A distinct type of data-driven dataset search has been implemented in SEEK [8], which finds datasets in which multiple user-provided genes show correlated expression patterns. This approach has the major advantage of not requiring differential expression analysis and can potentially be applied to any gene expression dataset. However, the implementation in SEEK is not very scalable and features only around 5000 datasets.

In this work, we present CORESH (coregulation search engine, https://alserglab.wustl.edu/coresh): a web server designed for querying public gene expression datasets based on a user-provided gene signature. CORESH ranks the datasets based on the level of coregulation of user-provided genes using a score inspired by principal component analysis (PCA), which can be applied to any gene expression matrix. Currently, CORESH operates on a compendium of 42 224 mouse and 44 253 human gene expression datasets from the GEO database, including datasets from both microarray and RNA-seq profiling. Further text mining coupled with enrichment analysis identifies terms overrepresented in the descriptions of top-ranked datasets, summarizing shared biological conditions. Individual datasets are linked to the corresponding GEO page, for more information on the dataset context, and to the Phantasus web application [9], where the gene expression changes in the dataset can be explored in more detail.

## Materials and methods

### Overview of CORESH search engine

CORESH is based on a dataset search pipeline previously described in [10]. The web server operates on a compendium of mouse and human gene expression datasets from the GEO database. Each dataset in the compendium is matched against the user-provided query gene set to calculate a relevance score inspired by PCA. The datasets are then ranked by this score and presented to the user. An additional text mining procedure is carried out to identify terms overrepresented in the descriptions of the top-ranked datasets, summarizing shared biological conditions.

### Dataset compendium

We use the GEO database as our primary data repository, which offers an extensive range of publicly available gene expression datasets. From this database, we use microarray and RNA-seq experiments from human and mouse. To obtain gene expression matrices, we use a pipeline previously described for the Phantasus web server [9], which complements microarray data available at GEO with RNA-seq counts from ARCHS4 [11] and DEE2 [12] databases.

Raw gene expression matrices are preprocessed to address the heterogeneity in platform technologies. First, we standardize gene identifiers to the Entrez format to facilitate consistent representation across all datasets. This step involves mapping platform-specific identifiers, such as probe identifiers for microarrays or Ensembl gene identifiers for RNA-seq data. Next, filtering and normalization of gene expression values are performed for each dataset in the compendium. For RNA-seq data, logCPM transformation is applied, while for microarray data, log transformation (if needed) and quantile normalization are used. For both data types, only the top 10 000 highly expressed genes are retained, and the gene expression values are then row-centered (with rows corresponding to genes). Finally, PCA-based dimensionality reduction is applied for large datasets to reduce the number of columns (samples) to 20.

In total, the CORESH compendium currently contains 86 477 curated experiments sourced from the GEO, ARCHS4, and DEE2 databases, divided into 44 253 human and 42 224 mouse experiments. Collectively, these experiments span over 2.5 million unique samples. The compendium undergoes regular updates to integrate newly available datasets.

### Dataset relevance score

**Figure 1.**
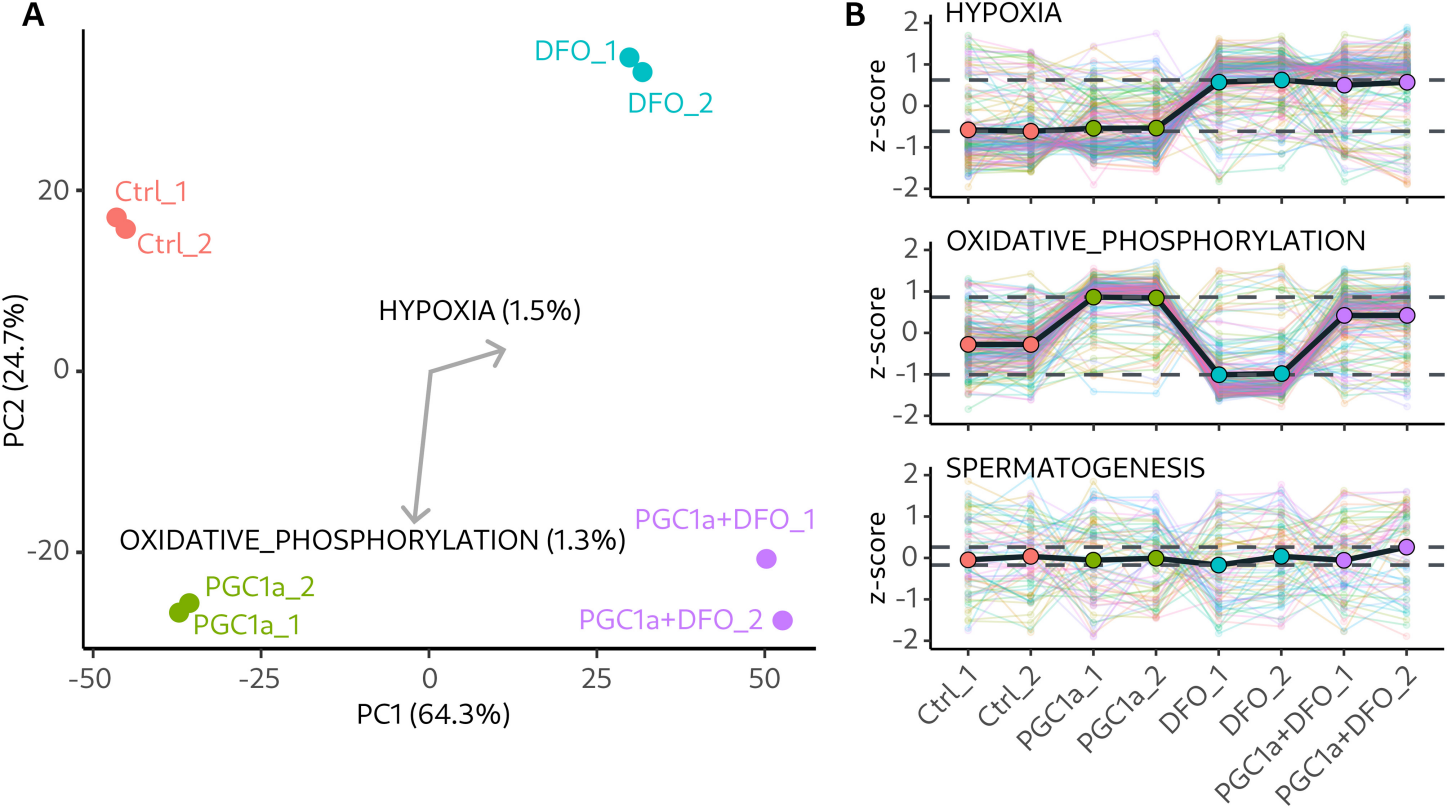
(**A**) PCA plot for dataset GSE42299. Points correspond to samples; arrows correspond to gene set directions. (**B**) Gene expression profiles of individual genes (thin lines) and averaged across the gene set (bold lines).

In CORESH, we introduce a comparison-independent score for ranking datasets from the compendium by their relevance to the query gene set. Inspired by PCA, this score captures the dataset variance explained by a certain direction in the sample space. Unlike PCA, which finds the optimal direction, our approach considers a single direction, defined by the query gene set. We quantify the variance of the sample projections onto this direction, which reflects the inter-gene correlation levels within the query gene set. Figure 1 illustrates this idea, using dataset GSE42299 as an example. There, direction along hypoxia gene set (from MSigDB hallmarks collection [13]) explains 1.5% of dataset variance and corresponds to treatment by deferoxamine. Similarly, oxidative phosphorylation gene set explains 1.3% of variance, but corresponds to overexpression of PGC-1α. High degree of correlation within these gene sets is evident in the gene set profile plots, whereas an unrelated spermatogenesis gene set has a flat profile (Fig. 1B).

More formally, our input consists of a gene expression matrix $E$ with $n$ rows (genes) and $m$ columns (samples), and a gene set $p$ of size $k$. We assume that the matrix $E$ is already preprocessed and that all rows are centered. We represent $p$ as a binary vector of length $n$ with $k$ ones at positions corresponding to the genes present in the query gene set. The percentage of variance explained by gene set $p$ can then be calculated as ${\mathrm{pctVar}}( {E,\ p} ) = \ \frac{{\|{{E}^T}p\|_2^2}}{{\|E\|_2^2k}} \cdot 100\% .$ This percentage can be used as a relevance score between the dataset and the query gene set.

In addition to calculating the percentage of explained variance, CORESH can assess its statistical significance. For this purpose, a *P*-value is calculated for each dataset: the probability of obtaining an explained variance equal to or greater than that of the query gene set using a random gene set of the same size. High efficiency of *P*-value calculation for many datasets is achieved by using an adaptive multilevel splitting Monte Carlo scheme, previously developed for fast gene set enrichment analysis method [14]. The implementation is available as geseca (gene set coregulation analysis) function in fgsea R package [14].

### Text-based metadata analysis

CORESH implements two types of text-based analysis to improve results interpretation: one at the dataset level and one for the overall ranking.

The dataset-level analysis aims to identify factors in the sample metadata that correlate with the query gene signature. Since many datasets lack well-structured sample metadata, we implemented a text-based approach. For each sample, the available metadata is converted into a bag-of-words representation. During processing of the user’s query, the query profile is calculated for each dataset by averaging the expression values for the genes in the query. This query profile is then tested for correlation against binary vectors that describe the presence or absence of words in the dataset samples’ metadata. The top positively and negatively correlated words are reported to the user.

The ranking-level analysis aims to summarize biological terms associated with the top-ranked datasets. The terms are extracted from dataset metadata, including the title, summary, and other fields, in a two-stage process. First, terms, potentially consisting of multiple words, are marked up in the original text using the scispacy package [15]. Then, the terms are expanded with the help of the GPT-4o-mini large language model and clustered together by similarity, an approach adapted from [16]. Finally, CORESH evaluates how frequently a given term appears in the top 300 datasets relative to its frequency in the entire ranked list using Fisher’s exact test.

### Web server architecture and interface

The CORESH web server is based on three integrated components: a front end, a back end, and a high-performance computing (HPC) environment. The front end is built in JavaScript using the React library and the Ant Design framework. The back end provides an HTTP-based API to power the React client and is written in Python using the Django framework. It handles utility tasks, such as gene identifier conversion using g:Profiler API [17], and manages submission of resource-intensive jobs to the HPC environment. The HPC environment carries out tasks such as calculating the percentage of explained variance for the query gene set and the corresponding *P*-value for each dataset in the compendium.

Currently, the CORESH implementation utilizes 10 processor cores per ranking task. This results in ranking times of under 10 s for the mode without *P*-value calculation, as tested on gene signatures from the CREEDS project [18] (Supplementary Fig. S1). In the mode with *P*-value calculation, ranking takes ∼1 min for queries of <100 genes and up to several minutes for queries with 500 genes. However, the actual response time also depends on the HPC cluster load and may exceed these estimates.

## Results

### CORESH web server

The CORESH web server allows users to search for gene expression datasets using a query gene signature. The input gene signature is typically derived from a gene expression experiment, e.g. a list of top upregulated genes in response to a treatment or markers of a specific cell population. The input form allows users to select the organism corresponding to the input gene signature, as well as the option to choose the database for the search query: human or mouse.

The main result of the CORESH search is a ranked list of publicly available GEO datasets where the input genes exhibit similar expression patterns; i.e. they show coordinated upregulation or downregulation across samples within those datasets. By default, the search engine ranks all datasets based on the percentage of variation explained by the direction in the sample space along the query gene signature. Additionally, an option to assess statistical significance is available, which can improve ranking, but requires more time for calculation.

**Figure 2.**
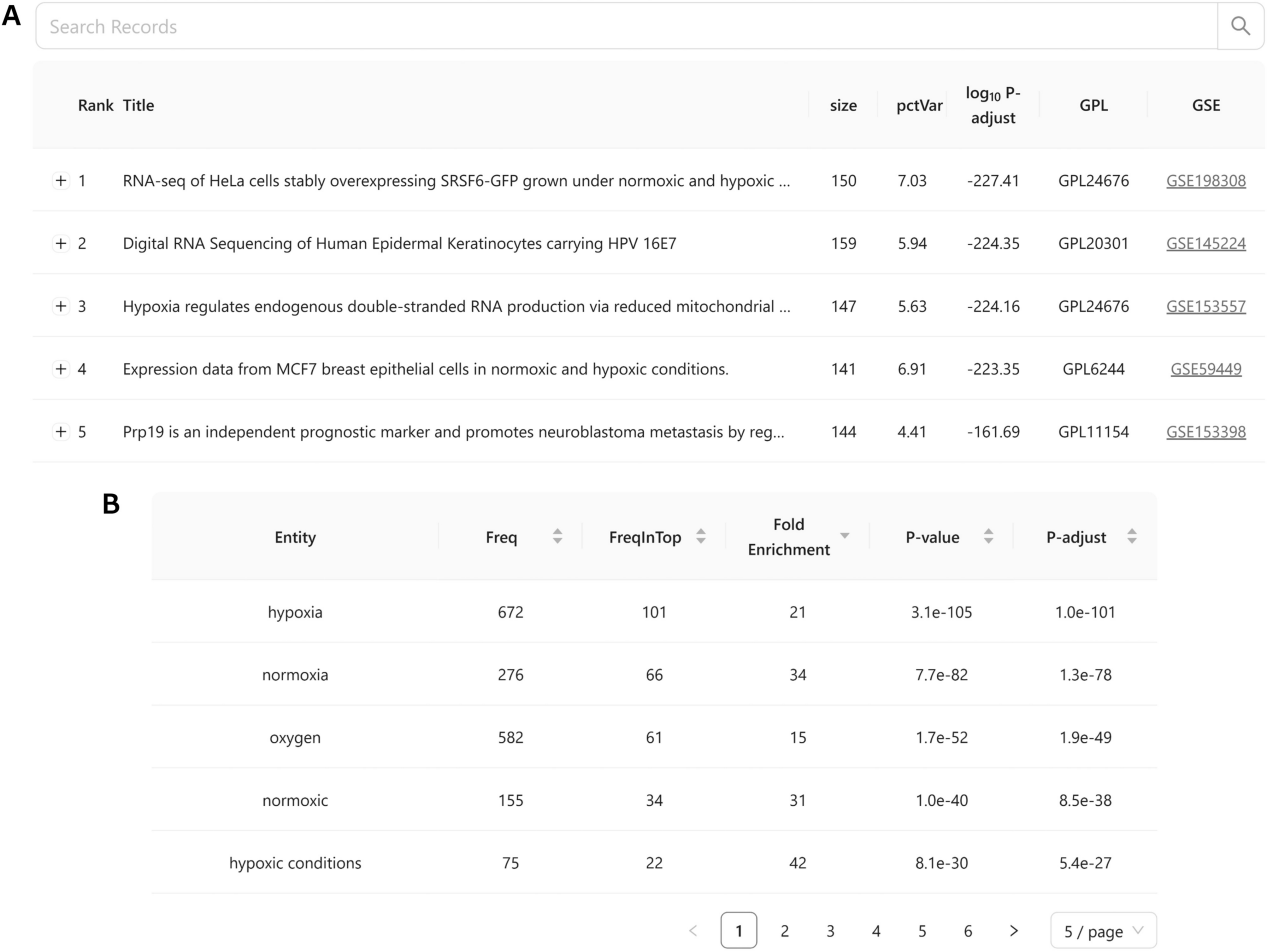
Screenshots of the search interface displaying search results for the hypoxia gene set from the MSigDB Hallmarks collection. (**A**) Interactive table showing the top five matched experiments for the gene set. (**B**) Table with the top five enriched terms.

The ranking is represented in an interactive table (Fig. 2A) that allows users to search and sort based on different parameters and fields (e.g. searching by GEO accession identifier, filtering datasets by keywords within available metadata, etc.). Each entry in the final table contains summary information in an expandable component, including, when available, sample metadata terms that correlate with the query gene set in the dataset. Additionally, a link to the Phantasus web application is provided, which can be used for further inspection of the corresponding dataset.

Another key component of the output is a table of terms enriched among the top-ranking datasets (Fig. 2B). This analysis summarizes the context where the query gene signature frequently appears and helps quickly identify major relevant biological processes and conditions.

### Case Study 1: gene signature induced by a pyroptosis secretome

In the first case study, we show how CORESH can be used for hypothesis generation. For this, we apply CORESH to investigate a gene signature induced in bone marrow-derived macrophages by a pyroptosis secretome, reproducing our previous results [10]. More specifically, in this study we considered a system where macrophages undergo pyroptosis without releasing IL-1β/α (denoted Pyro^−1^) and showed beneficial effects of Pyro^−1^ secretome on wound healing. Interested in understanding this effect, we profiled gene expression in macrophages treated with Pyro^−1^ secretome. We compiled a gene signature of genes upregulated in Pyro^−1^ treatment (Supplementary Table S1) and used this signature as a query for the search engine. Among the top hits, CORESH identified several datasets (e.g. GSE119509 and GSE41833) of macrophages treated with prostaglandin E2 (PGE2), where the Pyro^−1^ signature was upregulated following PGE2 treatment (https://alserglab.wustl.edu/coresh/load/example_pyroptosis; Supplementary Table S1 and Supplementary Fig. S2). This led us to hypothesize that PGE2 could mediate the effects of Pyro^−1^ treatment. Subsequent lipidomics analysis confirmed its enrichment in Pyro^−1^ supernatants, and further experiments confirmed the PGE2 wound healing effects *in vivo* [10].

### Case Study 2: smoking-associated gene signature

In another scenario, we demonstrate how CORESH can be used for the interpretation of a gene signature, either as a complement to or in place of classical pathway analysis. To illustrate this, we use a 20-gene signature obtained from asthma patient samples by the UnPaSt biclustering method [19] (Supplementary Table S2). In the study, the authors correlated this signature with the donors’ smoking status, which was available as a variable in their discovery cohort. Notably, when ran through commonly used gene set analysis tools like Enrichr [20], this signature cannot be directly connected to smoking—the closest gene set hits are NFE2L2-related reactive oxygen stress response gene sets. Similarly, there are no smoking-related datasets at the top of RummaGEO results (Supplementary Fig. S3). However, when used with CORESH, the top-ranked datasets are highly enriched in smoking-related experiments (https://alserglab.wustl.edu/coresh/load/example_smoking; Supplementary Table S2). This is reflected by term enrichment results, where the “smokers” is one of the top enriched terms, following “lung” and “lung cancer.” Furthermore, one can observe that the query signature is frequently correlated with smoking-related sample annotations (Supplementary Fig. S4). Finally, one can open the datasets in Phantasus, using the provided links, and visually confirm these associations (Supplementary Fig. S5).

## Discussion

Our study introduces the CORESH search engine, designed for signature-based search in publicly available gene expression datasets. CORESH extends the idea previously used in SEEK [8], where dataset relevance is scored based on the correlative behavior of the query genes within a dataset. The main advantage of this approach is that it does not require well-structured metadata and can be readily applied to any gene expression matrix. Consequently, this enables CORESH to work with a compendium of >86 000 gene expression datasets. This compendium covers most of the human and mouse datasets currently available either in GEO itself or in the RNA-seq quantification projects ARCHS4 and DEE2. We plan to regularly update the compendium to reflect the latest additions to these databases.

There are many scenarios in which CORESH can be useful. In Case Study 1, we demonstrate how it can help generate hypothesis about the mechanism of action of a treatment. Case Study 2 shows how it can complement classical pathway analysis, aiding in the interpretation of gene signatures. Beyond these examples, CORESH can be used to identify diseases relevant for a cell population using its marker genes from a single-cell RNA-seq study, to find potential phenotypes for drug treatment based on genes known to be regulated upon that treatment, or to explore cross-species conservation of gene programs by using human genes to identify corresponding mouse phenotypes and vice versa. This list is not exhaustive, and we expect many other applications to emerge.

In conclusion, the CORESH search engine simplifies discovery among tens of thousands of gene expression datasets using a gene signature as a query, and it has been proven useful for interpreting gene expression data in several published [10, 21] and ongoing studies.

## Supplementary Material

gkaf372_Supplemental_Files

## Data Availability

CORESH web server is freely available at https://alserglab.wustl.edu/coresh/. CORESH documentation is available at https://alserglab.github.io/coresh-docs/. An implementation of the CORESH core ranking algorithm is available at https://github.com/alserglab/coresh/ and https://doi.org/10.5281/zenodo.15256208; it uses a snapshot of the preprocessed dataset compendium available at https://www.synapse.org/coresh (Synapse project syn66227307). Gene signatures used in the case studies and the corresponding search results are available in the supplementary tables.
