# Supplementary material for: CORESH: a gene signature-based search engine for public gene expression datasets
Source: Nucleic Acids Res. 2025 May 5;53(W1):W187–92. doi: 10.1093/nar/gkaf372 (PMC12230675; doi:10.1093/nar/gkaf372)
Supplement: gkaf372_Supplemental_Files [file gkaf372_supplemental_files.zip › SupplementraryFigures.pdf]

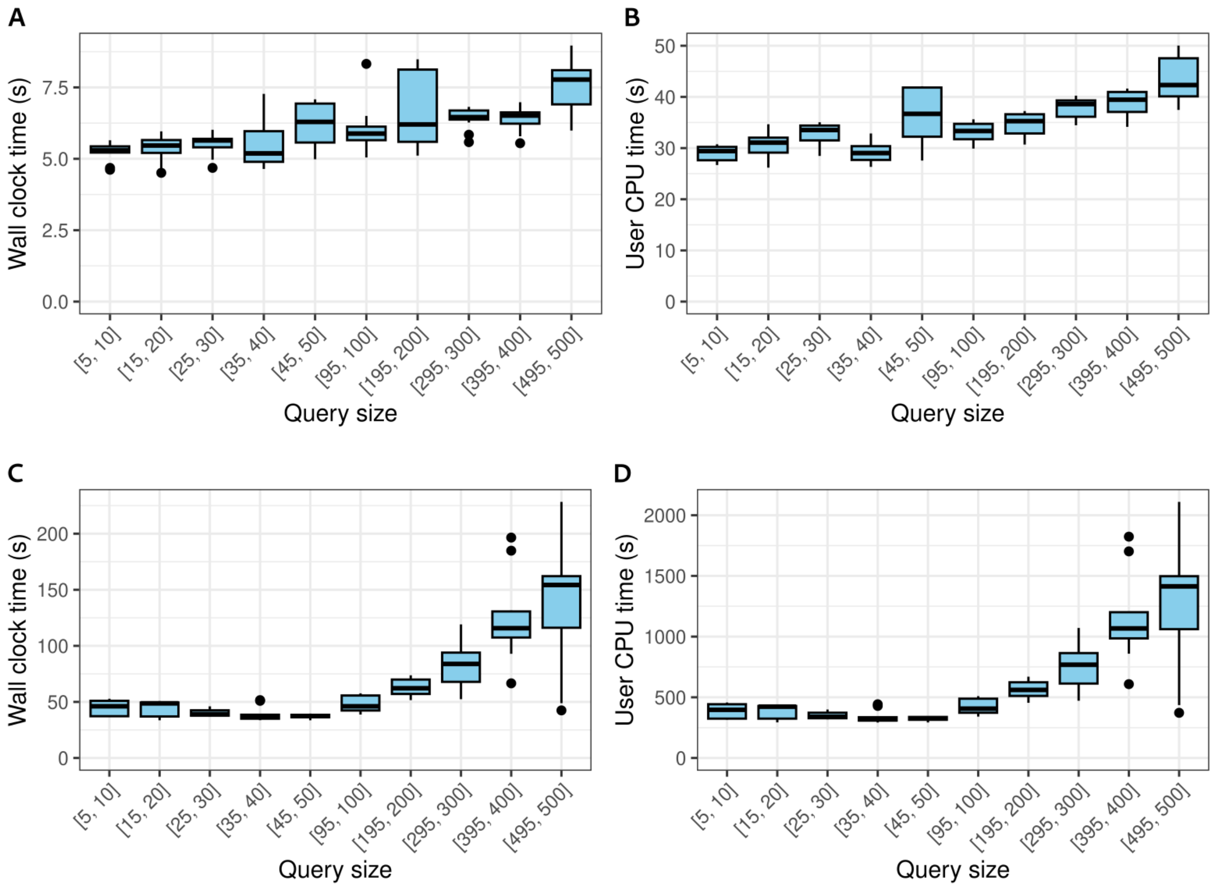

Figure S1. Ranking calculation times on a subset of CREEDS signatures for the mode without p-value calculation (A and B) and for the mode with p-value calculation (C and D).

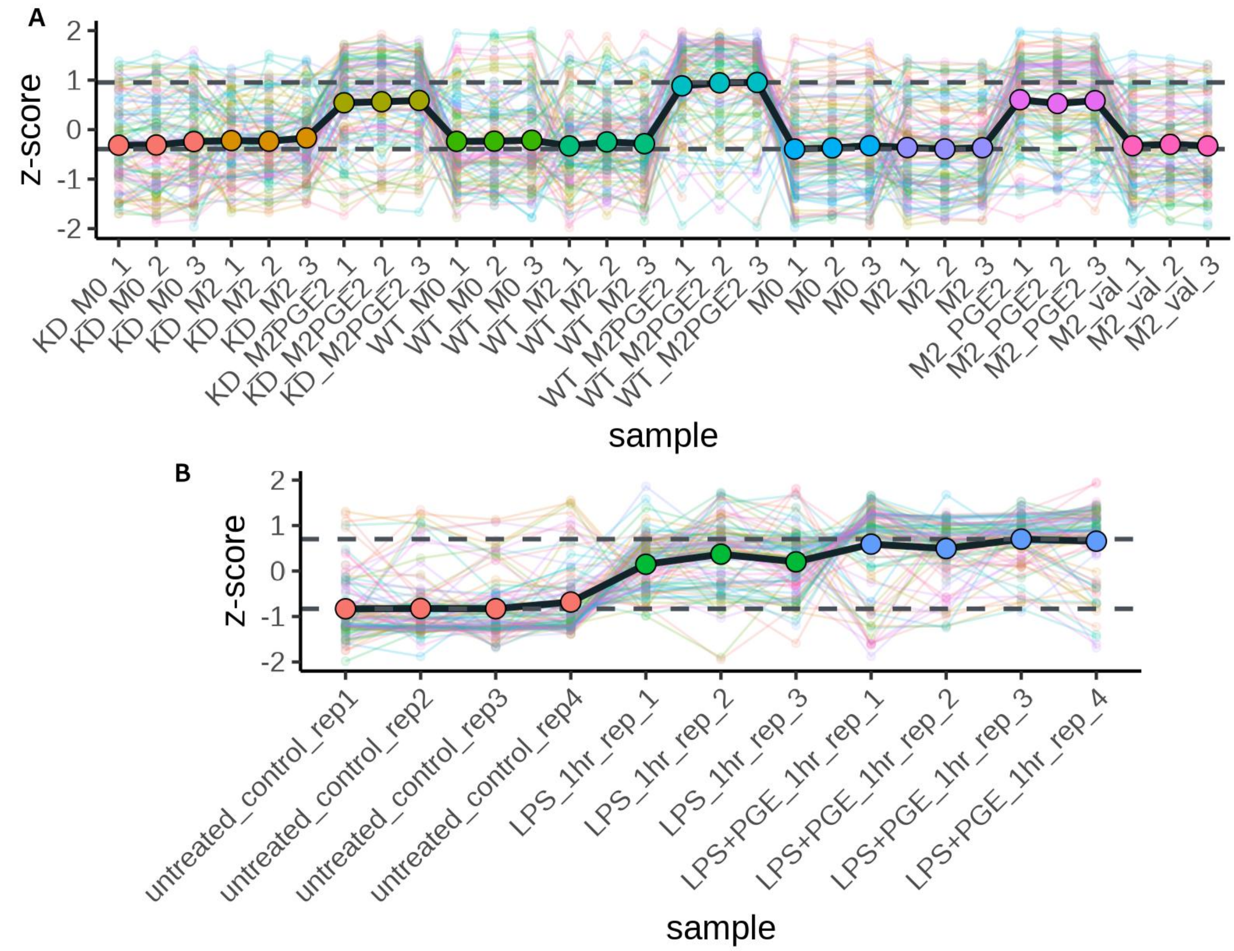

Figure S2. Profile of the Pyro^{-1} gene signature in datasets GSE119509 (A) and GSE41833 (B).

GEO Series	PMID	Title	Condition 1	Condition 2	Direction	Platform	Date	Gene Set Size	Overlap	Odds	PValue	Adj. PValue	Silhouette Score	Hypothesis	Enrichr Terms
GSE221916, GSE221917	37070193	PAXIP1 and STAG2 converge to maintain 3D genome architecture and facilitate promoter/enhancer contacts to enable stress hormone-dependent transcription [RNA-seq]	stag2ko dmso cell line a549 time 8 hours	nt 2 dexa cell line a549 time 8 hours	Down	GPL16791, GPL24676	2023-07-11	1550	14	28.1	1.12e-18	1.95e-13	-0.18		
GSE137912	31915379	Single cell RNA sequencing of cells treated with a KRAS G12C specific inhibitor	h358 lung cancer cells cell line ars1620	h2122 lung cancer cells cell line ars1620	Up	GPL16791	2020-01-17	815	12	45.8	2.67e-18	2.33e-13	0.52		
GSE124636	31145973	IRF2BP2 Modulates the Crosstalk between Glucocorticoid and TNF Signaling	rna seq hek293 gr sinon etoh cell line 1 transfection steroid depleted medium 72h 2 ethanol 6h	rna seq a549 siirf2bp2 etoh cell line 1 transfection steroid depleted medium 72h 2 ethanol 6h cells	Up	GPL11154	2019-06-03	1312	13	30.8	1.03e-17	5.99e-13	-0.26		
GSE238116	38287371	Effect of depletion or overexpression of EGR1 on gene expression in HCC cells (deletion in MHCC97H cells, overexpression in PLC/PRF5 cells)	mhcc97h cells parental biol rep cell line hepatocellular carcinoma wt	plc/prf5 cells egr1 overexpression biol rep cell line hepatocellular carcinoma	Down	GPL24676	2024-02-07	1399	13	28.9	2.36e-17	1.03e-12	0.74		
GSE171260, GSE171262	N/A	Protein neddylation as a therapeutic target in pulomonary and extrapulonary small cell carcinomas [FHSC39]	pdx tumor type human sclc fhsc39 vehicle derived	pdx tumor type human sclc fhsc39 mln4924 derived	Up	GPL16791	2021-04-01	274	9	102	8.74e-17	2.54e-12	0.41		
GSE110397	30573629	NK cell-mediated cytotoxicity contributes to tumor control by a cytostatic drug combination	a549 cell line lung	panc 1 cell line pancreatic cancer	Down	GPL16791, GPL17021	2018-12-01	1105	12	33.8	1.02e-16	2.54e-12	0.43		
GSE110397	30573629	NK cell-mediated cytotoxicity contributes to tumor control by a cytostatic drug combination	a549 cell line lung	panc 1 cell line pancreatic cancer	Down	GPL16791, GPL17021	2018-12-01	1105	12	33.8	1.02e-16	2.54e-12	0.43		
GSE86956, GSE86959	28255028	Genes regulated by SPDEF or FOXA3 in A549 lung carcinoma cells [RNA-seq]	a549 nkx2 1 pgk cell line background lung carcinoma cells /variation expressing infected control (pgk) lentivirus	a549 nkx2 1 foxa3 cell line background lung carcinoma cells /variation expressing infected lentivirus	Up	GPL16791	2017-03-02	1594	13	25.4	1.27e-16	2.60e-12	0.98		

1 2 ... 1639

Figure S3. Screenshot of RummaGEO results with the smoking-associated signature used as input.

	Rank	Title	size	pctVar	log_{10} P-adjust	GPL	GSE
−	1	Decreased Expression of Intelectin 1 in The Human Airway Epithelium of Smokers Compared to Nons...	10	1.51	-34.21	GPL570	GSE10006

Title: Decreased Expression of Intelectin 1 in The Human Airway Epithelium of Smokers Compared to Nonsmokers

Summary: Lectins are proteins present on cell surfaces or as shed extracellular proteins that function in innate immune defense as phagocytic receptors to recognize specific bacterial cell wall components. Based on the knowledge that cigarette smoking is associated with increased risk of bacterial infection, we hypothesized that cigarette smoking may modulate the expression of lectin genes in the airway epithelium. Affymetrix HG U133 Plus 2.0 microarrays were used to survey expression of lectin genes in large (3rd to 4th order bronchi) airway epithelium from 9 normal nonsmokers and 20 phenotypic normal smokers and small (10th to 12th order bronchi) airway epithelium from 13 normal nonsmokers and 20 phenotypic normal smokers. From the 72 lectin genes that were surveyed, there were no changes (>2-fold change, p<0.05) in gene expression in either large or small airway epithelium among normal smokers compared to nonsmokers except for a striking down regulation in both large and small airway epithelium of normal smokers of intelectin 1, a recently described lectin that participates in the innate immune response by recognizing and binding to galactofuranosyl residues in the cell walls of bacteria (large airway epithelium, p<0.003; small airway epithelium, p<0.002). TaqMan RT-PCR confirmed the observation that intelectin 1 was down-regulated in both large (p<0.05) and small airway epithelium (p<0.02) of normal smokers compared to normal nonsmokers. Immunohistochemistry assessment of biopsies of the large airway epithelium of normal nonsmokers demonstrated intelectin 1 was expressed in secretory cells, with qualitatively decreased expression in biopsies from normal smokers. Western analysis confirmed the decreased expression of intelectin 1 in airway epithelium of normal smokers compared to normal nonsmokers (p<0.02). Finally, compared to normal nonsmokers, intelectin 1 expression was decreased in small airway epithelium of smokers with early COPD (n= 13, p<0.001) and smokers with established COPD (n= 14, p<0.001), in a fashion similar to that of normal smokers. In the context that intelectin 1 is an epithelial molecule that likely plays a role in defense against bacteria, the down regulation of expression of intelectin 1 in response to cigarette smoking may contribute to the increase in susceptibility to infections observed in smokers, including those with COPD. Keywords: COPD

Positively correlated words: pack-years: 0.868, smoker: 0.51, gold-ii: 0.436

Negatively correlated words: non-smoker: -0.868

Explore gene expression data: https://alserglab.wustl.edu/phantasus/?geo=GSE10006

Figure S4. Screenshot of the detailed information for dataset GSE10006, one of the top datasets found for the smoking-associated signature.

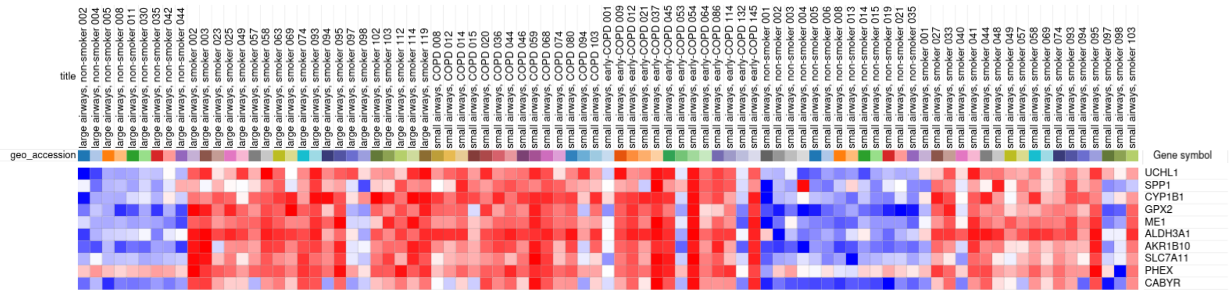

Figure S5. Screenshot of a Phantasus heatmap for dataset GSE10006 and genes from the smoking-associated gene signature. For the screenshot, the dataset was log-scaled and quantile-normalized; multiple probes for the same gene were collapsed into one; only the top 10,000 genes by average expression were retained.
